# Neuroimaging brain growth charts: A road to mental health

**DOI:** 10.1093/psyrad/kkab022

**Published:** 2021-12-30

**Authors:** Li-Zhen Chen, Avram J Holmes, Xi-Nian Zuo, Qi Dong

**Affiliations:** State Key Laboratory of Cognitive Neuroscience and Learning, Beijing Normal University, Beijing 100875, China; Department of Psychology, Yale University, New Haven, CT 06511, USA; Department of Psychiatry, Yale University, New Haven, CT 06511, USA; State Key Laboratory of Cognitive Neuroscience and Learning, Beijing Normal University, Beijing 100875, China; National Basic Science Data Center, Beijing 100190, China; Developmental Population Neuroscience Research Center, International Data Group/McGovern Institute for Brain Research, Beijing Normal University, Beijing 100875, China; Research Center for Lifespan Development of Mind and Brain, Institute of Psychology, Chinese Academy of Sciences, Beijing 100101, China; State Key Laboratory of Cognitive Neuroscience and Learning, Beijing Normal University, Beijing 100875, China

**Keywords:** growth chart, development, neuroimaging, reliability, mental health

## Abstract

Mental disorders are common health concerns and contribute to a heavy global burden on our modern society. It is challenging to identify and treat them timely. Neuroimaging evidence suggests the incidence of various psychiatric and behavioral disorders is closely related to the atypical development of brain structure and function. The identification and understanding of atypical brain development provide chances for clinicians to detect mental disorders earlier, perhaps even prior to onset, and treat them more precisely. An invaluable and necessary method in identifying and monitoring atypical brain development are growth charts of typically developing individuals in the population. The brain growth charts can offer a series of standard references on typical neurodevelopment, representing an important resource for the scientific and medical communities. In the present paper, we review the relationship between mental disorders and atypical brain development from a perspective of normative brain development by surveying the recent progress in the development of brain growth charts, including four aspects on growth chart utility: 1) cohorts, 2) measures, 3) mechanisms, and 4) clinical translations. In doing so, we seek to clarify the challenges and opportunities in charting brain growth, and to promote the application of brain growth charts in clinical practice.

## Introduction

Mental disorders, including but not limited to depression, anxiety, bipolar disorder, and schizophrenia, are common health concerns worldwide. According to the World Health Organization (WHO), the lifetime prevalence of mental disorders is high as 18.1–36.1% (Inter-Quartile Range; Kessler *et al*., 2007a). Beyond cognitive and psychological symptoms, individuals with mental disorders are also at increased risk of physical illness (Whiteford *et al*., 2013). More than 1 billion people globally were affected by mental and addictive disorders in 2016, which accounted for 7% of the global burden of disease (Rehm and Shield, 2019). Despite these staggering costs to both individual patients and our global community, the actual burden of mental disorders is likely underestimated, in part due to overlap between psychiatric and neurological diagnoses and the grouping of suicide and self-harm as a separate category (Vigo *et al*., 2016). Moreover, it is reasonable to speculate that the number of people suffering from mental disorders has increased in recent years due to the COVID-19 pandemic as well as political, economic, and climate crises (Khan *et al*., 2020; Boden *et al*., 2021).

While identifying mental disorders and their associated risks is of great significance for disease intervention and global burden relief, it is also challenging. In general, diagnosis of mental disorders is largely based on psychological evaluations, self-report questionnaires and surveys, or cognitive tests (e.g. processing speed and working memory tests). However, as highlighted by Philip Shaw (2016), psychiatric symptoms do not always associate with cognitive deficits. Psychological diagnostic approaches may not be sufficient in assessing the mental disorders, and more direct biologically focused measures are needed. As the term suggests, mental disorders are diseases of the mind, and for the past several decades there has been a concerted effort on the part of scientists to understand the neurobiological bases. Progress in this area suggests the incidence of various psychiatric and behavioral disorders is closely related to the atypical development of brain structure and function (Stoner *et al*., 2014; Kahn *et al*., 2015; Wakschlag *et al*., 2018). For example, abnormal structure and function in major cortical nodes of the salience network, a large-scale brain network theorized to support the detection and filtering of stimuli from the environment (Seeley *et al*., 2007; Power *et al*., 2011), have been observed as common neurobiological substrates across a broad spectrum of psychiatric disorders and their available treatments, especially the dorsal anterior cingulate cortex, anterior insula, and the cortico-striato-thalamo-cortical loop (Peters *et al*., 2016). Research on major depressive disorder showed disrupted network connectivity in the default mode network and the central executive network (Brakowski *et al*., 2017). Studies on patients with schizophrenia implicated frontal, temporal, and mesostriatal brain regions as key circuits in the development of positive, negative, and cognitive symptoms (McCutcheon *et al*., 2020).

The rates of many mental disorders increase during adolescence (Lee *et al*., 2014). Anxiety disorders like phobias and separation anxiety begin in childhood. Other anxiety disorders, including panic, generalized anxiety, and post-traumatic stress disorder, with onsets in the early teens (Kessler *et al*., 2005; Kessler *et al*., 2007b; Paus *et al*., 2008). Adolescence is a period of formative biological and social transition, which is sensitive for sociocultural processing (Nolen-Hoeksema and Girgus, 1994; Casey *et al*., 2008). During this period, multiple brain areas undergo both structural changes and functional reorganization (Blakemore and Mills, 2014). By measuring cortical thickness, the thickness of the grey matter ribbon surrounding the cortical sheet, and intracortical myelination of individuals aged 14–24 years, Whitaker *et al*. (2016) found that adolescent cortical myelination and shrinkage were coupled and associated with the synaptic-, oligodendroglial- and schizophrenia-related gene expression. Therefore, developmental variation of genetically patterned process of anatomical hubs may be relevant to cognitive and behavioral changes, as well as the high incidence of schizophrenia during human adolescence. A pattern of results which echoes prior work indicating that schizophrenia usually begins in late adolescence (Gogtay *et al*., 2011). Mental disorders with childhood or adolescent onsets tend to be more severe, undetected in the early stage, and may accrue additional co-morbid disorders (Paus *et al*., 2008). High-resolution neuroimaging provides an unprecedented window into the brain development, and can even provide reliable measurement of abnormal conditions before the appearance of clinical signs. If researchers can generate maps that link across diverse neural and cognitive states, clinicians will be able to leverage these discoveries to detect mental disorders earlier, perhaps even prior to onset, and treat them more precisely.

When it comes to the application of neuroimaging methods in the clinical practice of adolescent mental disorders, a major problem is the lack of population-level references that characterize normal brain development. A necessary step in the identification and study of atypical brain development is the establishment of growth charts for typical individuals in the population. Moreover, developmental trajectories can be used to detect the presence of sensitive periods and monitor the impact of environments and interventions on development (Di Martino *et al*., 2014a). While the research on the trajectories of healthy brain development is still in its infancy, researchers in this area can learn from the more than 200 years’ study of physical growth charts. Growth charts now provide detailed descriptions of the developmental process and velocity in a host of physical attributes, including height, weight, and head circumference across both groups and individuals. They are important tools in child health screening and pediatric clinical work-up, often conceptualized as a “road to health” (Cole, 2012). If growth charts of brain were available to the broader scientific and medical communities, studies focused on atypical brain development would gain standard references. It is conceivable that the efficiency of mental disorders diagnosis can be largely improved with the help of typical brain growth charts, which would be a great achievement in brain science. The construction of brain growth charts was limited by technologies in the past, but now the recent convergence of new imaging technologies and the increase of computational resources make it possible. Advances in Magnetic Resonance Imaging (MRI) provide opportunities to safely measure and map the structure and functional networks of human brain. Leveraging this approach, developmental neuroscientists have recently begun to establish brain growth charts (Dong *et al*., 2020), seeking to generate normative development curves across the lifespan (Ziegler *et al*., 2014; Marquand *et al*., 2016; Zuo *et al*., 2017; Reardon *et al*., 2018; Nobis *et al*., 2019; Bethlehem *et al*., 2021; Rutherford *et al*., 2021; Liu *et al*., 2021).

**Figure 1:**
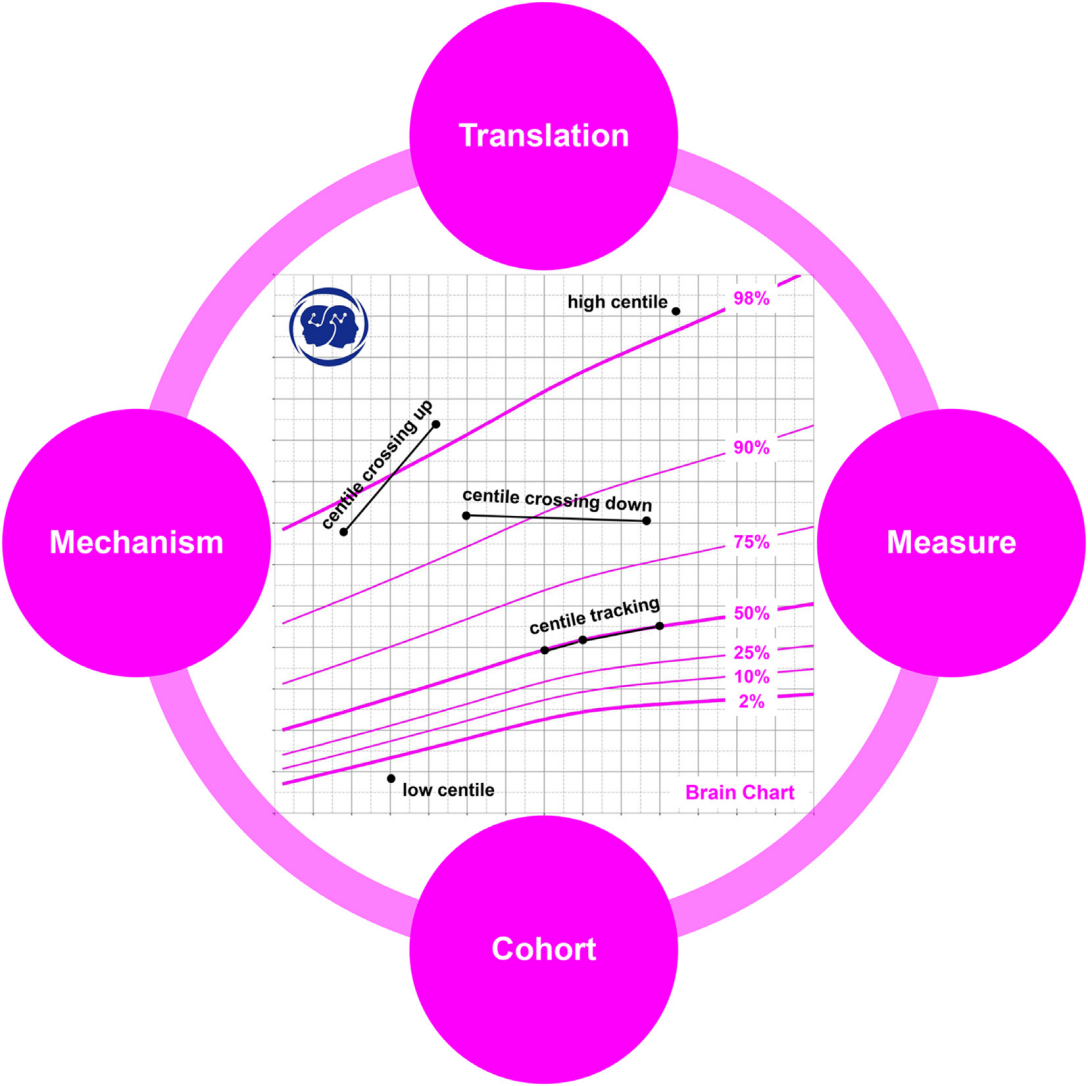
Brain growth charts and utilities. Key components of building a growth chart are illustrated as circles around a growth chart on brain volume. The four components (measure, cohort, mechanism, and translation) are seamlessly integrated into a growth chart model. A cohort is built to characterize individual differences in brain development across a certain age range (e.g. the school age). The cohort must include large enough and representative samples using reliable measures of the brain to achieve a valid growth chart. This chart can serve as a fundamental resource for mechanism discovery of brain development as well as its translations into clinical and educational conditions. A growth chart is also a ‘road to health’. The centered brain growth chart is developed based on the Chinese Color Nest Project cohort (Liu *et al*., 2021), consisting of a series of seven centile curves (98%, 90%, 75%, 50%, 25%, 10%, 2%) of brain volume for Chinese Han girls from childhood to adolescence (5–18 years old). Individual brain volume measurements can be expressed as centiles by plotting them on the chart. An individual centile indicates her brain volume, and the distance she has traveled along the growth road up to that age. It quantifies the volume/distance in terms of the centile (low versus high). The rate at which a girl grows is termed velocity and can be expressed either in measurement units (e.g. ml/year) for brain volume velocity, or alternatively in terms of centile change over time. The first form is the slope of the individual's growth curve on the chart, while, for the second, a growth curve that tracks along the centiles over time corresponds to average velocity, while if the curve crosses centiles up or down the individual is growing faster or slower than average. More details of the development of growth references and growth charts can be found in (Cole, 2012).

A host of modeling methods can be employed to chart brain growth, typical of which are Generalized Linear Mixed Modelling (GLMM), Generalized Additive Mixed Modelling (GAMM), and Generalized Additive Models for Location, Scale, and Shape (GAMLSS) (Rigby and Stasinopoulos, 2005; Zuur *et al*., 2009; Stasinopoulos *et al*., 2018), but the comparison of statistical models is not the purpose of this review, and detailed discussion can be found in previous literature (McArdle and Hamagami, 1992; Rigby *et al*., 2013; Anderson *et al*., 2019; Ramires *et al*., 2021). For brain growth chart research, the core aim is to build high-quality brain development cohorts of children and adolescents, quantify them reliably, and explore the corresponding developmental mechanisms. If successful, this work would allow for the establishment of reliable brain growth charts for clinical application. In the present paper, we review the recent progress in the development of brain growth chart from four key aspects including 1) cohorts, 2) measures, 3) mechanisms, and 4) translations of brain development (see Fig. 1 for a general framework). In doing so we seek to clarify the challenges and opportunities in developmental neuroimaging as we build towards a thorough understanding of brain development.

## Cohorts

Brain growth charts must be built on data from representative samples. Primary growth data and related information of WHO child growth standards 2007 comes from 8 440 individuals with diverse ethnic and cultural backgrounds, including Brazil, Ghana, India, Norway, Oman and the United States (USA) (WHO, 2009). The reference data of USA growth charts built by Centers for Disease Control and Prevention (CDC) comes from five big national survey data sets (Kuczmarski *et al*., 2002). In China, the commonly used development reference standard in clinical practice is derived with the data of 94 302 healthy individuals aged 0–19 in nine cities which represent north, central, and south China (Li *et al*., 2009). The selection of representative samples is a crucial and challenging problem. The UK Biobank, a population-based cohort, collects extensive phenotypic and genotypic detail from over 500 000 participants (Sudlow *et al*., 2015). However, the representativeness of the UK Biobank cohort was questioned as Fry and colleagues reported a “healthy volunteer” selection bias in UK Biobank (Fry *et al*., 2017). For sampling in typical development studies, it is necessary not only to extend sample size, but also to select individuals strategically. For example, control the female/male ratio, recruit participants with diverse family economic and parents’ educational levels from diverse areas, since these factors may potentially affect the trajectories of individual development (Hanson *et al*., 2013; Ingalhalikar *et al*., 2014; Noble *et al*., 2015).

Another major consideration for the establishment of growth charts is their longitudinal nature. While cross-sectional research only yields age-related individual differences across a population rather than the dynamic development processes within an individual, longitudinal studies can help to circumvent common developmental inferential errors derived from cross-sectional data (Baltes, 1968; Kraemer *et al*., 2000; Howell *et al*., 2019). Despite include some cross-sectional data, a key component for the construction of WHO child growth standards is a longitudinal cohort, in which children were examined in a sequence of 21 visits starting at birth and ending at 24 months of age which allowed the detection of growth velocity (WHO, 2009). The recommended international fetal growth standards by INTERGROWTH-21^st^ Project for the clinical interpretation of routinely taken ultrasound measurements are also built on the basis of fetal longitudinal study following the prescriptive WHO approach (Papageorghiou *et al*., 2014; Ohuma *et al*., 2021).

Therefore, it is important for developmental neuroscientists to recruit representative participants and gather data from them repeatedly over an extended period when constructing growth charts, including brain growth charts. Several large-scale and multi-modal neuroimaging cohorts that are suitable for the construction of brain growth charts have been built (Volkow *et al*., 2018; Liu *et al*., 2021), table 1 reflects a non-exhaustive list of typical brain development cohorts from prenatal to young adults. These cohorts cover a diverse set of healthy individuals and reflect important contributions for the construction of brain growth charts, explorations of developmental underpinnings, as well as genetic and environmental factors that may influence health across the lifespan. Since the longitudinal nature is preferred in the research of brain growth charts, the following briefly introduces several representative longitudinal development cohorts from different countries/regions, which involve different study designs, extensive age range, and various development measurements.

**Table 1:** A non-exhaustive summary of typical brain development cohorts from prenatal to young adults.

Longitudinal	Starting Age	Follow-up Duration	Country	Website and/or References
Generation R	Prenatal	From 2002*	Netherlands	https://generationr.nl (Hofman *et al*., 2004; White *et al*., 2013)
GUSTO	Prenatal	From 2009*	Singapore	https://www.gusto.sg (Soh *et al*., 2013)
FinnBrain	Prenatal	From 2010*	Finland	https://sites.utu.fi/finnbrain (Karlsson *et al*., 2018)
HBCD	Prenatal	10 years	USA	https://www.nimhd.nih.gov/programs/collab/HBCD-study
YOUth-B&C	Prenatal	6 years	Netherlands	https://www.uu.nl/en/research/youth-cohort-study (Onland-Moret *et al*., 2020)
DCHS	Prenatal	5 years	South Africa	http://www.paediatrics.uct.ac.za/scah/dclhs (Donald *et al*., 2018)
CBD	6–12 years	1 year	China	(Fan *et al*., 2021; Lei *et al*., 2021)
devCCNP	6–18 years	2.5 years	China	http://deepneuro.bnu.edu.cn/?p=163 (Yang *et al*., 2017; Liu *et al*., 2021)
cVEDA	6–23 years	1 or 2 years	India	https://cveda-project.org (Sharma *et al*., 2020; Zhang *et al*., 2020)
YOUth-C&A	8–10 years	6 years	Netherlands	https://www.uu.nl/en/research/youth-cohort-study (Onland-Moret *et al*., 2020)
ABCD	9–10 years	10 years	USA	https://abcdstudy.org (Barch *et al*., 2018; Casey *et al*., 2018; Volkow *et al*., 2018)
Dev-CoG	9–14 years	3 years	USA	http://devcog.mrn.org (Stephen *et al*., 2021)
NCANDA	12–21 years	3 years	USA	http://www.ncanda.org/index.php (Brown *et al*., 2015)
IMAGEN	14 years	From 2010*	UK Germany France Ireland	https://imagen-europe.com (Schumann *et al*., 2010; Mascarell Maričić *et al*., 2020)
Cross-Sectional	Age Range	Subject N	Country	Website and/or References
PING	3–20 years	1493	USA	(Jernigan *et al*., 2016; Taquet *et al*., 2021)
HBN	5–21 years	10 000	USA	https://healthybrainnetwork.org (Alexander *et al*., 2017)
NKI-RS	6–85 years	> 1000	USA	https://fcon_1000.projects.nitrc.org/indi/pro/nki.html (Nooner *et al*., 2012)
PNC	8–21 years	1445	USA	https://www.med.upenn.edu/bbl/philadelphianeurodevelopmentalcohort.html (Calkins *et al*., 2015; Satterthwaite *et al*., 2016)
Mixed	Age Range	Subject N	Country	Website and/or References
BCP	0–5 years	∼ 500	USA	https://www.humanconnectome.org/study/lifespan-baby-connectome-project (Howell *et al*., 2019)
NIH Pediatric	0–18 years	∼ 500	USA	(Evans, 2006; Almli *et al*., 2007; Walker *et al*., 2016)
HCP-D	5–21 years	> 1300	USA UK	https://www.humanconnectome.org/study/hcp-lifespan-development (Harms *et al*., 2018)

*Note*. The design classification is only applicable to neuroimaging measurements. Accelerated longitudinal cohorts are classified as longitudinal cohorts, and all mixed cohorts have clear cross-sectional and longitudinal portions. Asterisk indicates that this is an ongoing cohort, and how long the follow-up would last is not clear.

The Generation R Study, started from 2002, is a prospective population-based cohort study that is designed to identify environmental and genetic factors of growth and health from the fetus to early adulthood with an integrated epidemiological, clinical and basic research approach (Hofman *et al*., 2004). A total of 9 778 mothers with a delivery date from April 2002 to January 2006 were enrolled. General follow-up rates of children until 4 years old exceed 75% (Jaddoe *et al*., 2007; Jaddoe *et al*., 2010). Since 2009, 6–8-years old children from the Generation R Study were invited to participate MRI scanning (White *et al*., 2013). And all children and their parents were re-invited when children were 10, 13 and 16 years old (Kooijman *et al*., 2016). In 2017, a new cohort study “Generation R Next” was launched. The Generation R Study ubiquitously captures the growth and development of children. MRI scanning allows scientists to investigate developmental neurobiological trajectories of children and to explore the relationship between atypical development and physical and/or psychological health, provides a comprehensive dataset for developmental neuroscience community. For instance, by investigating data from the Generation R study, Muetzel *et al*. (2018) reported that higher baseline ratings for psychiatric symptomatology could predict smaller increases in both subcortical gray matter volume and global fractional anisotropy over time but the reverse relationship did not hold. Therefore, future neuroimaging studies should additionally explore the possible downstream effects of psychopathology on the brain, instead of being limited to explaining the brain differences observed in psychopathology as an underlying neurobiological substrate. Similar long-term longitudinal development cohorts have been built by other countries, such as the Growing Up in Singapore Towards healthy Outcomes (GUSTO) cohort (Soh *et al*., 2013; Qiu, 2020) and the FinnBrain cohort (Karlsson *et al*., 2018). Different from the Generation R study, these cohorts imaged children longitudinally from birth.

The Adolescent Brain Cognitive Development (ABCD) study is the largest longitudinal study of adolescent brain development and health in the USA. It aims to establish a unique database of adolescent brain and cognitive development, tease apart the biological and environmental factors that influence or alter developmental trajectories and to answer the pressing public health questions about adolescent development (Barch *et al*., 2018; Volkow *et al*., 2018). The ABCD study plans to recruit 10 000 participants aged 9–10 years across 21 sites in the USA for at least 10 years of follow-up. All participants received comprehensive measurements of brain development, health biomarkers, cognitive function, substance abuse, family, and environmental factors. MRI was used to assess human brain function and structure development every two years (Casey *et al*., 2018). Baseline tests of ABCD have yielded some important discoveries. For instance, resting state functional connectivity patterns predict individual-differences in neurocognition (Sripada *et al*., 2020), brain activations found in the task functional MRI (fMRI) (Chaarani *et al*., 2021) are consistent with the published literature, and brain structure have incremental validity for associations with psychopathology in youth (Mattoni *et al*., 2021). With the progress of ABCD study, it is possible to demystify the longitudinal changes during adolescent brain development and corresponding regulatory factors, and chart typical brain development trajectories across the second decade in life. Despite the valuable nature of these data, interpretive issues have arisen regarding aspects of the ABCD baseline brain function study (Chaarani *et al*., 2021). In brief, the reported effect sizes in the ABCD functional tasks show subtle correlations with preformance on those tasks, which may damage the validity (see also Kennedy *et al*., 2021). While the cause of this issue is not yet clear, it illustrates the importance of experimental design and assocaited analytic approaches in developmental neuroimaging, calling for a unfied measurement theory across multiple disciplines. A point we elaborate on in the *Measures* section.

Different from the single longitudinal design, the Chinese Color Nest Project (CCNP) is an accelerated longitudinal cohort which tracks individuals at different initial ages and enables the detection of a larger age range in a shorter period of time. CCNP aims to accumulate psychological, behavioral and brain imaging data from 6–90 year-old Chinese and hence to establish the norm of the Chinese human brain. CCNP comprises three phases: devCCNP, matCCNP and ageCCNP. As the development component of CCNP, the devCCNP has been implemented in two cities (Chongqing and Beijing), and it would be carried out in more areas of China in the future. Started in 2013, devCCNP has successfully acquired CCNP-Southwest University (CCNP-SWU) samples. All participants aged between 6–18 years. The follow-up period of each participant is 30 months after the start of the study, including MRI scans and cognitive behavior tests at three time points (baseline, follow-up 1 and follow-up 2). The follow-ups were carried out in the 15th and 30th month respectively (Yang *et al*., 2017). Following the same study design, this project is continuing in Beijing currently (Liu *et al*., 2021). Using data from devCCNP, Dong *et al*. (2020) established age-normed brain templates for children and adolescents at one-year intervals and the corresponding growth charts. They also compared the brain templates and growth charts between Chinese and USA school-age participants and indicated that brain growth standards are, in part, ethnicity dependent. Furthermore, Zhou *et al*. (2021) charted the amygdala developmental curves of children and adolescents in devCCNP and compared different segmentation measurements with the manual tracing method. These two studies have important implications in promoting the methodology development of the reliability and validity in brain development research, which are detailed in the next section.

## Measures

To build a powerful brain growth chart and apply it for clinical auxiliary diagnosis, the prerequisite is to ensure that the selected measurements are valid. In the measurement of individual difference, reliability is a necessary condition for validity (Xing and Zuo, 2018). Reliability is a relative metric involve both the between-subject and within-subject variability. When the variability within subjects is smaller than that between subjects, it is easier to identify different individuals, and makes the measurement more reliable (Xing and Zuo, 2018; Zuo *et al*., 2019). The range of reliability varies largely in different fields. By contrast, the reliability coefficient of WHO anthropometric measurements for constructing the growth charts including length, height and arm circumference is above 95% (Group, 2006), while the reliability of general neuroimaging measurements with fMRI is considerably lower (Zuo and Xing, 2014). The low-reliability problem in neuroimaging has been overlooked in the past decades. With the increasing demand for the transformation of neuroimaging research results into clinical practice, there is a growing consensus that reliability should be a primary concern. In order to achieve high reliability of human brain measurement, the measurement targets, tools, and metrics should be considered.

The average statistical power of neuroscience research is low, which reduces the probability to detect true effect (Button *et al*., 2013). In determinations of statistical power, reliability, sample size, and effect size interplay with each other. When the reliability of MRI metrics is limited by available methods, a large sample size is required to enhance the statistical power (Zuo *et al*., 2019). However, heavy financial burden and the public bias on the safety have limited the sample size of MRI research studies (Poldrack *et al*., 2017). Fortunately, neuroscientists have made a lot of efforts in popularizing open science, which make it easier to obtain the large sample of MRI data. Here, pioneering attempts include aggregating MRI data from laboratories around the world (Biswal *et al*., 2010) or building large-scale national projects and carrying out associated data sharing, such as the ABCD study (https://abcdstudy.org), Human Connectome Project (HCP) (https://www.humanconnectome.org), and UK Biobank (https://www.ukbiobank.ac.uk). Specially, HCP contains the test-retest dataset for the research on reliability (Van Essen *et al*., 2013). The Consortium for Reliability and Reproducibility (CoRR), initiated in 2014, was built to establish test-retest reliability as a standard for methods development in human brain connectome. Typical resting state fMRI data from 18 international sites were opened freely, enabled researchers to estimate reliability and reproducibility (Zuo *et al*., 2014). Promisingly, Granville J. Matheson (2019) proposed a method that can make better use of results from previously published test-retest studies to enhance the reliability of new studies. Transferring this method to neuroimaging research will greatly improve the use efficiency of test-retest datasets, and the future research designs will be better guided.

In a previous commentary (Xing and Zuo, 2019), we pointed out “to do a valid job, we must make tools reliable first”. “Tools” include the approaches investigators use to estimate reliability and the methods to collect and analyze data. Improper use of reliability assessing method may result in misleading conclusion. Intra-Class Correlation (ICC) (Shrout and Fleiss, 1979) is one of the most frequently used methods to evaluate reliability in neuroimaging which quantifies the ratio of within-subject variance to across subject variance. The interpretation of ICC must follow the strict Gaussian assumption. However, in practice, some researchers may overlook this prerequisite. A recently proposed method, named discriminability, defined as the fraction of frequency at which the similarity across-subject measurements is smaller than similarity within-subject measurements, can assess the reliability of multivariate data more flexibly and be used to any stage of data processing (Wang *et al*., 2020). This method is based on nonparametric energy statistics (Rizzo and Székely, 2016) and kernel mean embeddings (Muandet *et al*., 2016) approaches, and it is equivalent to ICC under the Gaussian assumption for univariate data (Wang *et al*., 2020; Bridgeford *et al*., 2021; Milham *et al*., 2021). Methods of data collection and analysis can contribute to low reliability in neuroimaging studies. Taking MRI as an example, anatomical MRI (aMRI), fMRI, and diffusion MRI (dMRI) form the common modalities of MRI, and their reliabilities are quite different. Usually, results of aMRI and dMRI studies have higher reliability as their metrics have explicit structural basis in macro or micro level. Prior work on the evaluation of brain morphology's test-retest reliability indicate almost perfect reliability of thickness, gyrification and fractal dimensionality measures (Madan and Kensinger, 2017), and dMRI metrics have also been shown high stability in neonates, healthy adults and chronic stroke patients (Boekel *et al*., 2017; Merisaari *et al*., 2019; Lewis *et al*., 2020). Conversely, the reliability of fMRI remains a concern. Meta-analyses have revealed poor overall reliability of fMRI-derived seed connectivity and common task activation measures, suggesting a long way to go for the implementing translations of the common task-fMRI measures into brain biomarker discovery or individual-difference research (Noble *et al*., 2019; Elliott *et al*., 2020).

Many factors can undermine the reliability and validity of MRI studies. For example, the low-frequency physiological phenomena may influence the signal of resting-state fMRI, and after correcting for the pressure of end-tidal CO_2_ fluctuations, the reproducibility of the resting-state fMRI measures improved significantly (Golestani *et al*., 2017). The toolboxes, algorithms or templates used for data preprocessing and analysis can also introduce errors. Zhou *et al*. (2021) observed systematic distinctions in amygdala volumes between manual and automatic segmentation approaches. Regarding manual segmentation as the “gold standard”, FreeSurfer estimated larger amygdala, volBrain underestimated the amygdala volume, and FSL demonstrated a mixed pattern. Accordingly, it is perhaps unsurprising that growth trajectories of amygdala built by different methods exhibited different shapes. Nuisance regression during data preprocessing can result in extra nuisance (Hallquist *et al*., 2013; Chen *et al*., 2017). When bandpass filtering of resting state fMRI data is followed by nuisance regression of unfiltered signals, the functional connectivity would be inflated systematically (Hallquist *et al*., 2013). Further more, Dong and colleagues (2020) confirmed the difference in morphological characteristics and volumetric growth between the Chinese and USA children across school ages. These data highlight the necessity of both age-specific and ethnicity matched brain templates to account for population-level shifts in developmental trajectories. Different metrics in different space have diverse reliabilities too. Generally, the reliability of surface-based (2D) metrics are higher than that of volume-based (3D) metrics (Ghosh *et al*., 2010; Tucholka *et al*., 2012). Independent component analysis with dual regression, local functional homogeneity, and functional homotopic connectivity are more reliable than degree centrality, eigenvector centrality, and functional connectivity (Zuo and Xing, 2014).

How can we conduct more reliable functional connectomics studies? Comparing the measurement reliability in functional network neuroscience systematically, Jiang *et al*. (2021) suggested that using a whole brain parcellation to define network nodes, constructing functional connectome in multiple slow frequency bands, optimizing topological economy of networks, and characterizing information flow are highly desirable for the reliable individual difference measurement. In addition, experimental psychology and neuropsychology approaches may not suitable for the direct study of individual differences in brain function (Hedge *et al*., 2018). Elliott *et al*. (2021) proposed four critical strategies for the reliability enhancement of fMRI, including “extended aggregation, reliability modeling, multi-echo fMRI (ME-fMRI), and stimulus design”. Specifically, researchers should 1) prolong the scanning time in studies as reliability can be greatly improved by increasing scanning time (Birn *et al*., 2013; Taxali *et al*., 2021); 2) develop reliable mathematical models for specific brain functions, such as models to predictive or discriminate special disorder-related brain features (Marquand *et al*., 2016; Wolfers *et al*., 2018; Marquand *et al*., 2019; O'Muircheartaigh *et al*., 2020; Taxali *et al*., 2021); 3) utilize multiple echoes to separate nuisance physiological signal from the signals of interest, including head motion, cardiopulmonary physiology, or other types of imaging artifact (Lombardo *et al*., 2016; Kundu *et al*., 2017); and 4) optimize the stimulus and design new fMRI tasks which can measure individual difference, instead of using traditional group-targeted tasks (Elliott *et al*., 2020).

Last but not least, large-scale cohorts are usually established by pooling of multi-site data. However, inconsistent platforms will introduce systematic differences that distort the image information, lead to spurious results, and thus damage reliability severely (Focke *et al*., 2011; Chen *et al*., 2014). Even when standardizing protocols and image acquisition parameters are used, the site effects cannot be totally removed (Glover *et al*., 2012). Several statistical models have been put forward to control site effects (Jovicich *et al*., 2006; Chen *et al*., 2014; Fortin *et al*., 2017). ComBat, a technique adopted from batch-effect correction in genomics, performs great in removing unwanted inter-site variability in multi-modal MRI data, including dMRI maps (Fortin *et al*., 2017), cortical thickness (Fortin *et al*., 2018), as well as functional connectivity measurements (Yu *et al*., 2018). Other attempts at reducing site effects include the improved ComBat method (Yamashita *et al*., 2019; Maikusa *et al*., 2021) and meta-analytic approach (Koshiyama *et al*., 2020). With the development of new study frameworks and methodology, we can imagine a future in which highly reliable laboratory neuroimaging results are used to construct brain growth charts and guide clinical practice.

## Mechanisms

Brain growth charts can quantify and display human neurodevelopmental patterns. Understanding mechanisms behind the patterns and subsequent functional changes would provide core insights into healthy brain development. The trajectories depicted by neuroimaging show marked changes during brain development. In the first 2 years of life, cortical grey-matter volume increases robustly, cortical thickness peaks during this period and decreases thereafter, the growth of cortical white-matter volume is comparatively slower. Reaching adolescence, grey-matter volume tends to decrease, whereas white-matter volume continues to increase (Gilmore *et al*., 2018). These changes are widely read as results of synaptic pruning and myelination of axons during ontogeny. Synaptogenesis occurs concurrently with dendritic and axonal growth and myelination. The number of synapses in auditory cortex reach their peaks before the prefrontal cortex, where the increase extends to adolescence (Huttenlocher and Dabholkar, 1997). This trend is highly consistent with findings that primary somato/sensory and visual regions mature before higher-order association areas (Gilmore *et al*., 2018). The density of mouse in neocortex, measured by electron micrographs, tend to increase with cortical thickness (Schüz and Palm, 1989). More importantly, as discussed above, there is a significant correlation between mental disorders and atypical features of brain development, while the occurrence of mental disorders in adolescence is connected with abnormal synaptic pruning (Keshavan *et al*., 1994; Germann *et al*., 2021). Taken together, these studies imply that synaptic pruning may underlie aspects of typical and/or atypical brain morphological development.

However, it must be noted that these interpretations should be considered with caution because they are still controversial. Paus and colleagues (2008) given some insightful explanations. They argued that the change of synaptic density is unlikely to affect the cortical volume or thickness as, in non-human primates, synapses reflect a very small fraction of the cortical volume (Rakic *et al*., 1986). Rather, they attributed changes in grey matter to myelination of intra-cortical fibers, since it may result in the change of grey matter proportion in T1 image. Besides, the increase of white matter volume is more likely caused by the change in axonal caliber than the increase of myelination degree. While the precise links across distinct features of brain development are yet to be established, a path forward may emerge with the aid of new technologies. Positrons Emission Tomography (PET) imaging of synapses, for instance, has great potential. Several PET radiotracers have been developed that allow for *in vivo* synapses visualization and quantification, enable the detection of synaptic density in rodent, non-human primate, and human brains (Finnema *et al*., 2016; Becker *et al*., 2020). The combination of PET and MRI may pave the way for a systematic interpretation of brain development mechanisms.

In the process of development, the network architecture of brain undergoes profound changes in conjunction with shifts in underlying anatomical features. The homogeneity and physical distance are key determinants of interregional connectivity strength in different species’ brain including drosophila, mouse, macaque monkey, and human (Goulas *et al*., 2019). The empirical connectomes increase with the improvement of homogeneity, while the long distance may weaken the connectivity strength. Similar results were obtained in our previous work, which applied the generative network models to lifespan development (Zuo *et al*., 2017). In this work, we also found a shift from anatomically driven (distance) to nonspatial generative rules (homogeneity) of brain organization across the lifespan. The connectome of young brains tends to be limited by the distance between regions. With the increase of age, the weight of nonspatial factor increases. Mental disorders may disrupt this topological rule. Study on schizophrenia patients with childhood-onset revealed decreased strength of functional connectivity over short distances in patients, and hence the global mean connection distance was significantly larger than that of normal individuals (Alexander-Bloch *et al*., 2013).

Studies targeted at interareal connectivity changes in the brain indicated that the organization of cerebral cortex follows certain patterns (Margulies *et al*., 2016; Haak *et al*., 2018; Huntenburg *et al*., 2018). Margulies *et al*. (2016) discovered a principal gradient in human brain, which is anchored from primary sensory/motor regions to transmodal association regions and may explain how cognition arise from the topographical organization of large-scale functional networks. A recent review (Sydnor *et al*., 2021) supports the existence of principal gradient at macrostructural, microstructural, functional, metabolic, transcriptomic, and evolutionary levels of analyses. Intriguingly, cortical development from childhood to adolescence approaches the gradient architecture observed in adults. Unimodal cortices reach their mature positions early in development, while high order association cortex matures later. Primary gradients in the neonatal connectome runs between sensorimotor and visual anchors (Bernhardt *et al*., 2020). Evidence from Dong *et al*. (2021) indicated a shift of gradients between childhood and adolescence. In children, similar with neonates, the gradient was anchored within the unimodal cortex. In participants who have reached the age of adolescence, the principal gradient transitions into an adult-like spatial mode, in which the primary cortex is anchored at one end and the transmodal cortex at the other end. For individuals with mental disorders, there could be some atypical connectivity transitions between sensory and higher-order default mode regions relative to typically-developing individuals (Hong *et al*., 2019). The protracted development of the association cortex gives rise to an extended window of plasticity, also makes them preferentially vulnerable to environmental or other negative factors (Buckner and Krienen, 2013; Sydnor *et al*., 2021), which could be the potential reason why many mental disorders are associated with impaired higher-order cortex and emerge during adolescence.

## Translations

The establishment of brain growth charts is only the beginning of the research on brain development emphasized in this review. The ultimate purpose is to understand the genetic and environmental factors that modulate brain development, help the diagnosis of mental disorders, and finally serve the health policy to better brain development.

There are two common strategies for the study of atypical brain development trajectories. The first strategy is to screen the individuals with mental disorders from common large-scale cohorts, which are not specially built for psychiatric studies. In addition to the longitudinal cohorts introduced earlier, several cross-sectional cohorts can be used, such as the National Consortium on Alcohol and Neuro Development in Adolescence (NCANDA) (Brown *et al*., 2015), the Pediatric Imaging, Neurocognition, and Genetics Data Repository (PING) (Jernigan *et al*., 2016), and the Philadelphia Neurodevelopmental Cohort (PNC) (Calkins *et al*., 2015). The age range of individuals in these large-scale cohorts is suitable for the study of adolescent mental disorders. Importantly, these cohorts include both healthy and abnormal individuals, which can be classified into none, mild, moderate, and severe groups according to the psychosis spectrum. In this case, brain charts of different disease processes can be explored dynamically. By referring to the normative brain development trajectory, investigators can not only identify patients with mental disorders from healthy individuals, but also predict the severity stratification (Gur *et al*., 2014). What's more, these cohorts collected bio-samples for genetic analysis, which can assist the further examination of epigenetic changes. A series of studies leveraged data from the PNC cohort are best examples of identifying atypical developmental trajectories in psychiatric patients with the help of growth charts. An early study showed that individuals with psychotic symptoms were neurocognitively delayed across the age range and such delay was related to symptom severity (Gur *et al*., 2014). Kessler *et al*. (2016) found that intrinsic connectivity networks of individuals with attention-deficit/hyperactivity disorder (ADHD) displayed a “shallow maturation” pattern when compared with the normal brain growth trajectory. Despite these discoveries, we cannot ignore the cross-sectional nature of these cohorts. Utilizing longitudinal cohorts, there is a chance to provide more compelling evidence about the brain growth charts’ profit to detect mental disorders. Jalbrzikowski *et al*. (2019) combined data from a longitudinal study and three cross-sectional cohorts including PNC, illustrated that connectivity of amygdala with many regions (prefrontal cortices, striatum, occipital cortex, and thalamus) represented a downward trend with age in normative trajectories, but this phenomenon could not be observed in youth with psychosis spectrum disorders. Similarly, Shaw *et al*. (2018) conducted a study using longitudinal data from four cohorts to characterize the growth trajectories of the cerebellum, described a slow first and fast afterwards growth trend of cerebellar white matter in ADHD patients.

Another strategy is to establish the cohorts related to specific mental disorders, from which we can directly explore the related developmental patterns. The Brazilian High Risk Cohort Study (BHRC) is a longitudinal study that follows the individuals with high risk of suffering mental disorders (ADHD, anxiety disorders, obsessive compulsive disorder, psychosis, and learning disorders) and tries to identify the developmental trajectories and causal pathways for these diseases (Salum *et al*., 2015). EU-AIMS Longitudinal European Autism Project (LEAP) is a largescale, multi-center, multi-disciplinary observational cohort on biomarkers for autism spectrum disorder (ASD) (Charman *et al*., 2017). With the aid of this cohort, Zabihi *et al*. (2019) demonstrated highly individualized patterns of deviations in cortical thickness in ASD patients and these deviations are correlated with severity of repetitive behaviors and social communicative symptoms. A similar dataset, the Autism Brain Imaging Data Exchange (ABIDE), shares MRI data and phenotypic information of individuals with ASD (Di Martino *et al*., 2014b). Based on this dataset, a brain age prediction model found smaller brain age in ASD patients, suggesting the delayed brain development of these patients (Wang *et al*., 2021). What's more, the datasets have been used to construct the age-dependent computer aided diagnosis system for ASD (Haghighat *et al*., 2022). Reasonably, these methods can be extended to other mental disorders to decode the atypical brain development trajectories related to psychiatry (Wolfers *et al*., 2018; Wolfers *et al*., 2019).

Regardless of the study strategy, the description of atypical growth patterns is inextricably linked with the need to create reference maps of normative developmental trajectories. An extraordinary work (Bethlehem *et al*., 2021) recently aggregated MRI data from more than one hundred thousand healthy and clinical individuals in over 100 cross-sectional and longitudinal studies. The age of individuals ranges from 115 days post-conception to 100 years postnatal years, reflecting the largest ever dataset of lifespan development. Brain growth charts were modeled based on this large-scale dataset, and several novel brain developmental milestones were identified. Importantly, new patterns of neuroanatomical differences across typical neurological and psychiatric disorders emerging during development were revealed (e.g. ASD, ADHD, and anxiety disorders). However, as the authors suggested, the growth charts built in their work are more suitable for the use of scientific research rather than clinical utility because of the relatively limited use of longitudinal imaging in the dataset and methodological issues associated with combining multiple datasets which may blur the underpinning of brain development. The availability of MRI technology, the accumulation of longitudinal research data and the advance on developmental population cognitive neuroscience would speed up the application of brain growth charts in clinical practice in the future.

## Concluding Remarks

We reviewed the prevalence of mental disorders and the relationship between mental disorders and atypical brain development. In this context, the significance of typical brain growth charts for clinical reference was emphasized. In view of how to build reliable brain growth charts, cohorts, measures, and underpinnings of brain development were discussed. We also introduced the translational applications of brain growth trajectories in clinical studies and practice about mental disorders.

In the *Cohorts* section, we mainly introduced three large-scale development cohorts. As listed in table 1 and Liu *et al*. (2021), there are many other neuroimaging cohorts that are applicable for the research on brain development. Review on the brain development measurements revealed the severity of the low reliability problem faced by MRI research. In particular, it posed a great challenge to task fMRI. The importance of appropriate templates, toolboxes, preprocessing procedures, and metrics in future data analysis was pointed out. Other considerations like different machines, scanning protocols, magnetic field strength, and study sites can also influence the reliability of MRI studies. How to minimize the adverse effects of these factors on reliability is still a topic of intense study.

The exploration of brain developmental mechanisms would help us understand typical and atypical development better. We reviewed the brain development at cellular level and macro functional network level, discussed the debates on the role of synaptic pruning in brain morphological changes, and put forward a promising method to solve controversies. The changes in neurochemical transmission and the regulation of genetics could be potential developmental mechanisms as well. For instance, changes in hormone level have a substantial impact on brain structure and function (Vijayakumar *et al*., 2018; Gracia-Tabuenca *et al*., 2021). Barendse *et al*. (2020) observed that increased testosterone levels over time were related to increases in white matter cross-section in the inferior fronto-occipital fasciculus in adolescence. During development, genes like microcephaly make a chief contribution to the enlargement of the human brain (Gilbert *et al*., 2005). Genome-wide association studies of brain imaging phenotypes also demonstrated the widespread genetic architecture of brain (Elliott *et al*., 2018). Development is influenced by genetics, stochastic processes, environment, and culture (Thompson and Moreno, 2018). These factors may act on abovementioned mechanisms, change the brain development trajectories, promote or hinder brain health and thereby lead to better behavioral performance or symptoms of psychiatric illness.

Finally, although this review takes mental health as a starting point to introduce the implication of typical brain growth charts, the range of “growth charts” can further extend to lifespan, and the utility of brain growth charts should not be limited to mental health, they are also meaningful for other aspects of brain health (e.g. educational or neurological conditions). Brain growth charts can be valuable under educational settings for monitoring different levels of normal developing across school ages, and understanding the individual differences in brain and mind development during the educational implementation. It would provide evidence-based supports for teaching activities, motivating more scientific policy-making in education. Studies based on the developing human connectome project (dHCP) provides important insights for newborn brain development (Ciarrusta *et al*., 2020; Fenchel *et al*., 2020; Eyre *et al*., 2021). When comparing premature infants’ brain microstructure to term neonates, researchers found strikingly heterogeneous deviations from typical development in preterm infants, and greater deviations were associated with more extreme prematurity and predicted poorer cognitive abilities (Dimitrova *et al*., 2020; O'Muircheartaigh *et al*., 2020). Ageing has been proved to be the primary factor of most irreversible neurodegenerative diseases (Hou *et al*., 2019). Nobis *et al*. (2019) illustrated that the rate of hippocampal volume loss would speed up once arriving middle age. As the measurement of hippocampal volume has been proved useful to diagnose and track progression in Alzheimer disease (Schuff *et al*., 2009; Feng *et al*., 2021), it is meaningful to depict the change trajectory of hippocampus in middle-aged and elderly people, and the same hold true for other brain regions. Several cohorts have been launched to detect the development course and brain mechanisms of neurodegenerative diseases and seek feasible treatments for them, e.g. Pre-symptomatic Evaluation of Experimental or Novel Treatments for Alzheimer Disease (PREVENT-AD) cohort (Breitner et al., 2016; Tremblay-Mercier *et al*., 2021), the Open Access Series of Imaging Studies (OASIS) (Marcus *et al*., 2007; Marcus *et al*., 2010), and Beijing Aging Brain Rejuvenation Initiative (BABRI) (Yang, C *et al*., 2021; Yang, Y *et al*., 2021). Once established typical brain development charts across the lifespan, these cohorts would function better for the understanding of atypical brain ageing. Early detection and effective treatment of diseases would be possible with the help of brain development charts.
